# SAMSA2: a standalone metatranscriptome analysis pipeline

**DOI:** 10.1186/s12859-018-2189-z

**Published:** 2018-05-21

**Authors:** Samuel T. Westreich, Michelle L. Treiber, David A. Mills, Ian Korf, Danielle G. Lemay

**Affiliations:** 1 Genome Center, University of California, Davis, California USA; 2 Department of Food Science and Technology, University of California, Davis, California USA; 3 USDA ARS Western Nutrition Research Center, Davis, CA USA

**Keywords:** Metatranscriptomics, Metagenomics, Pipeline, Software, Bioinformatics, Open access, Annotation, Functions, Metatranscriptome, RNA-seq, Cluster, GALAXY, Tool, Microbiome, Bacteria, SAMSA

## Abstract

**Background:**

Complex microbial communities are an area of growing interest in biology. Metatranscriptomics allows researchers to quantify microbial gene expression in an environmental sample via high-throughput sequencing. Metatranscriptomic experiments are computationally intensive because the experiments generate a large volume of sequence data and each sequence must be compared with reference sequences from thousands of organisms.

**Results:**

SAMSA2 is an upgrade to the original Simple Annotation of Metatranscriptomes by Sequence Analysis (SAMSA) pipeline that has been redesigned for standalone use on a supercomputing cluster. SAMSA2 is faster due to the use of the DIAMOND aligner, and more flexible and reproducible because it uses local databases. SAMSA2 is available with detailed documentation, and example input and output files along with examples of master scripts for full pipeline execution.

**Conclusions:**

SAMSA2 is a rapid and efficient metatranscriptome pipeline for analyzing large RNA-seq datasets in a supercomputing cluster environment. SAMSA2 provides simplified output that can be examined directly or used for further analyses, and its reference databases may be upgraded, altered or customized to fit the needs of any experiment.

## Background

High-throughput sequencing methods are now used to identify both culturable and unculturable microbial species. Although 16S ribosomal profiling is still most commonly used, researchers are adopting more comprehensive sequencing methods such as metagenomics and metatranscriptomics. Metagenomics—sequencing of all DNA from a diverse sample—reveals which microbes are present. Metatranscriptomics—sequencing of all RNA from a diverse sample—captures all gene expression, giving a view of which microbes are active and what they are doing. Despite the power of metatranscriptomics, there are still relatively few bioinformatics tools designed to handle this complex type of data.

The first metatranscriptomics analysis papers described workflows but did not provide code or a software program [1, 2]. Web-based tools, such as MG-RAST [3] or COMAN [4], enables users to analyze metatranscriptome data, although the level of analysis possible is limited. Although these approaches allow analysis without the need for local compute resources, both MG-RAST and COMAN are dependent upon a service that may become oversubscribed and slow, and they do not support mapping to custom reference databases [4]. Another recent tool that is not a web service, MetaTrans [5], relies on rRNA for organism identification, necessitating a lack of biological ribodepletion and thus reducing the level of captured functional data from mRNA. Our previous work demonstrated that approximately 50 million paired-end reads per sample are needed to accurately quantify transcripts in stool samples [6] when the total RNA has been ribodepleted. For un-ribodepleted samples, the majority of RNA will be rRNA, so the sample would require hundreds of millions of reads for accurate functional quantification. We also previously demonstrated that ribodepleted RNA has biases, and so cannot be used for quantification [6]. Finally, some recent metatranscriptome pipelines (anvi’o, [7], IMP, [8]) rely on BLAST [9] for annotations, which may be unacceptably slow when processing multiple millions of sequences per sample.

Simple Annotation of Metatranscriptomes by Sequence Analysis (SAMSA) was the first open-source bioinformatics pipeline designed specifically for metatranscriptomic data [6]. SAMSA was built for researchers with minimal bioinformatics experience and who may not have a supercomputing cluster available for their use. SAMSA worked in conjunction with MG-RAST [3], the aforementioned public annotation service capable of handling metagenomic or metatranscriptomic data. SAMSA improved upon MG-RAST by offering local preprocessing and paired-end merging of RNA sequences, uploading to MG-RAST, downloading annotations, and analyzing the results. One downside of MG-RAST is that the software and database are not under control of the user. For maximum reproducibility, one must have complete control over all parameters, and this is not possible with a web service.

SAMSA2 is entirely standalone and is designed for cluster computing. The software and databases can be completely containerized for reproducibility. SAMSA2 uses DIAMOND [10] for aligning sequences, which greatly increases its speed relative to BLAST or public services. SAMSA2 handles end-to-end analysis of metatranscriptomes from quality control of sequencing reads through publication-ready images. SAMSA2 is packaged with documentation and sample data.

## Implementation

### Recommended sequencing parameters

To provide accurate measurements of gene expression occurring within the complex and varied environment of a microbiome, it is important to obtain sufficient depth of sequencing, along with reads of length necessary to avoid erroneous alignments. Longer reads are generally more advantageous, as they are less likely to be erroneously aligned to references. The SAMSA pipeline includes a merging step to combine overlapping paired-end reads; although this step can be skipped if using single-end samples, the shorter read lengths can contribute to incorrect annotations if they are not at least 150 bp. For paired-end reads, it is also recommended that sequencing libraries be produced from cDNA size selected to ensure overlaps of the two reads in the paired-end format.

In previous work [6], we established that between 10 and 20 million annotations—which translates to 40–50 million raw (unannotated) reads in stool metatranscriptomes—ensuring the best results when estimating the abundance of transcripts and obtaining stable measures of gene expression. Given the high abundance of ribosomal RNAs (rRNAs) naturally present in total RNA extracted from microbiome samples, ribodepletion is strongly encouraged. A digital ribodepletion step is included in the SAMSA pipeline, but physical ribodepletion before sequencing ensures that a higher number of mRNAs are sequenced. Ribodepletion kits do not effectively remove all rRNA from samples, but they greatly reduce (> 80%) the number of sequenced rRNA reads that must later be discarded from bioinformatic analysis. In summary, it is recommended that metatranscriptomes be ribodepleted and sequenced in a paired-end format of at least 100 bp with a minimum of 40 million reads per sample.

### Tool dependencies and version control

SAMSA2 uses several recognized tools, including PEAR [11] and Trimmomatic [12] for preprocessing and read trimming, SortMeRNA [13] for filtering of ribosomal RNAs, and DIAMOND for annotation [10]. For version control, and to ensure compatibility and reproducibility, binaries of these tool dependencies can be downloaded and unpacked using a single install script, install_packages.bash, which is provided in the setup folder with the SAMSA2 installation.

### Preprocessing

**Fig. 1:**
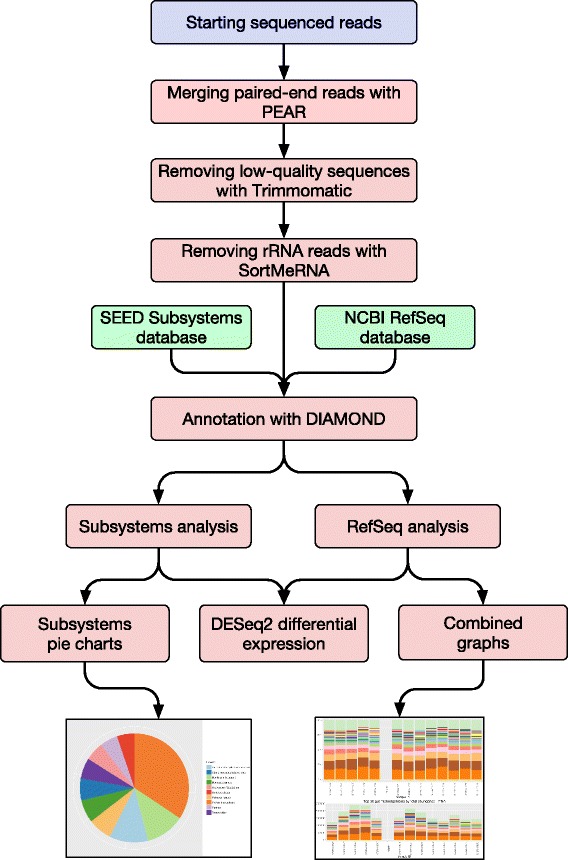
The SAMSA2 analysis pipeline. Starting sequence reads are merged, cleaned, and filtered to remove ribosomal RNA (rRNA) sequences. At the annotation step, DIAMOND can be used to incorporate any custom database as an annotation reference. Results are condensed and analyzed using custom Python scripting, and saved as standard data tables that can be imported into R to generate figures or for statistical comparison

An overview of the flow of data through the SAMSA2 pipeline is in Fig. 1. The first step in the SAMSA2 pipeline is to merge paired-end files if this sequencing type was used. PEAR is a fast and efficient paired-read merger [11] that can be installed as a precompiled binary. When used on two paired-end files, it creates a merged output containing all reads with overlap, as well as two “notCombined” files that contain forward and reverse unassembled reads, respectively. Generally, only the merged reads are used in the remainder of the pipeline, although if a high number of reads cannot be merged, it may be advisable to use both the merged and the forward “notCombined” read sets to ensure that an adequate total number of annotations is obtained.

Following paired-end read merging (if necessary), low-quality sequences and/or adaptor contamination are removed. Trimmomatic [12] is a flexible read trimmer designed for Illumina sequence data, which can both remove low-quality sequences, and identify and remove any potential adaptor contamination. Trimmomatic produces a cleaned output file that is ready for the next step in preprocessing.

Finally, although the majority of ribosomal sequences are hopefully removed from the metatranscriptome before via ribodepletion prior to sequencing, SortMeRNA [13] provides another digital sweep, ensuring that ribosomal reads are removed before annotation. Removal of ribosomal reads at this step reduces the total number of sequences that will be annotated, increasing downstream speed and reducing file sizes. Another reason to discard these sequences is that the remaining ribosomal reads cannot be used to assess organism activity because the ribodepletion is biased towards certain organisms [6]. SortMeRNA installs with several reference rRNA databases for both bacterial and eukaryotic ribosome sequences. SortMeRNA outputs both identified ribosomal sequences and sequences that do not match against known ribosomes; these “other” sequences are carried forward to the annotation step in SAMSA2.

### Annotation

SAMSA2 uses DIAMOND [10], a superfast BLAST-like algorithm, to perform annotations against one or more selected reference databases. DIAMOND is designed specifically for annotating large numbers of read sequences against a reference database simultaneously, with speeds up to 10,000 times faster than traditional BLAST.

Before input sequences can be annotated by DIAMOND, reference databases must be created. Instructions for creating DIAMOND-searchable databases are included in the documentation provided with SAMSA2, along with links, and more detailed installation instructions for creating databases from NCBI’s RefSeq database [14] and the SEED Subsystems hierarchical database [15] for functional activities. It is possible to add other databases, such as the Carbohydrate Active Enzyme (CAZy) database [16]. After a database has been converted into a DIAMOND-searchable binary, experimental sequence files are annotated against this reference.

### Aggregation and downstream processing

Annotated files returned from DIAMOND searches are returned in plaintext; each sequence from the infile that was matched with a sequence in the reference database occupies one line in the outfile. The next step is to aggregate these large, line-by-line files into a condensed, sorted summary of output.

Using Python, SAMSA2 converts the DIAMOND output into a sorted abundance count, creating two files—one file is for organism annotations, whereas the other file contains functional annotations of the same reads. When reads are annotated against a hierarchical database such as SEED Subsystems, this sorted abundance count also includes all levels of hierarchy information.

These sorted abundance counts are saved as the final step of the automated workflow for the SAMSA2 pipeline. These count files can be viewed directly to identify most active organisms or functions within a metatranscriptome and are also used by R in the final step of the SAMSA2 pipeline for statistical analysis.

The final step in the SAMSA2 pipeline is analysis using R scripting for calculating differential expression and generating tables and graphs, treating the sorted abundance counts from the aggregation step of the SAMSA2 pipeline as input. Calculation of significant differences at both the organism and the function level for each database is performed automatically in the batch submission script, whereas the outputs may be imported into an interactive R session for further analysis or figure generation.

**Fig. 2:**
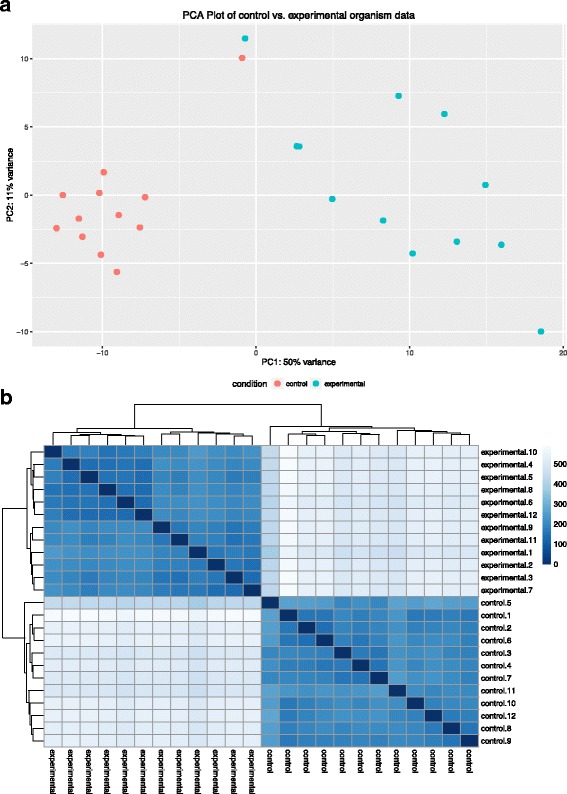
**a** PCA and **b** heatmaps generated by SAMSA2 visualization scripts. Comparisons can be made using either organism or functional annotation results, or based on any other incorporated database. Both plots show how similar whole metatranscriptomes are to each other. Greater similarity is associated with **a** closer dots in the PCA plot or **b** darker blue color in the heatmap

**Fig. 3:**
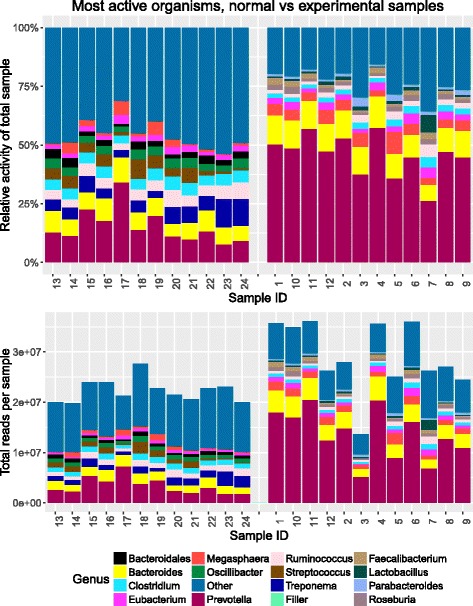
Example stacked bar plot. SAMSA2’s default stacked bar graph shows both relative (top) and absolute (bottom) transcript counts per genus with samples grouped according to control or experimental metadata designations

**Fig. 4:**
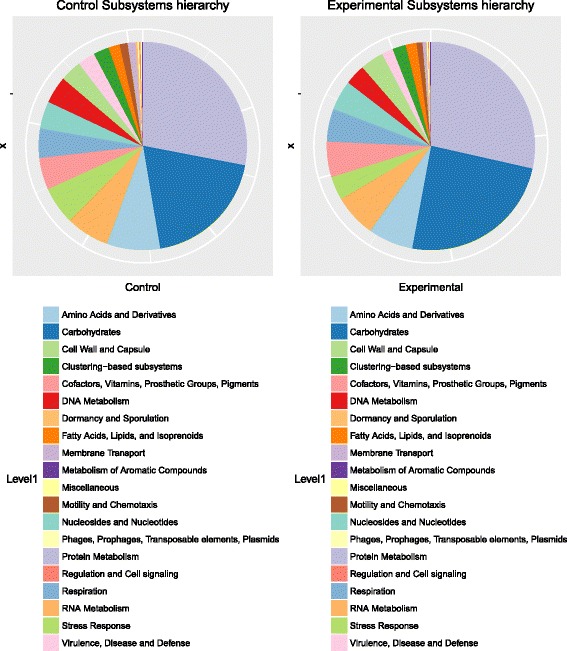
Example SEED Subsystems annotation pie charts at hierarchy level 1. Pie charts or other figures can be generated for every level of SEED Subsystems hierarchy

Multiple R scripts are included with the SAMSA2 pipeline, each designed to carry out a single analysis action on the processed data. These included R scripts can perform the following actions: determine statistically significant differentially active organisms or functions between two groups of samples (such as experimental samples compared with normal controls), calculate microbial diversity statistics for each metatranscriptome, create PCA plots based on organism or functional activity (Fig. 2a), create heatmaps of organism or functional activity (Fig. 2b), and create stacked bar graphs for each metatranscriptome, displaying the most abundant functions or organisms within each metatranscriptome (Fig. 3). For metatranscriptomes annotated against the Subsystems hierarchical database, included R scripts can identify statistically significant differentially expressed categories at each hierarchy level, and can create pie charts for each metatranscriptome to show relative activity matching each functional activity category (Fig. 4).

## Results

### Improved speed and accuracy for metatranscriptome analysis

**Fig. 5:**
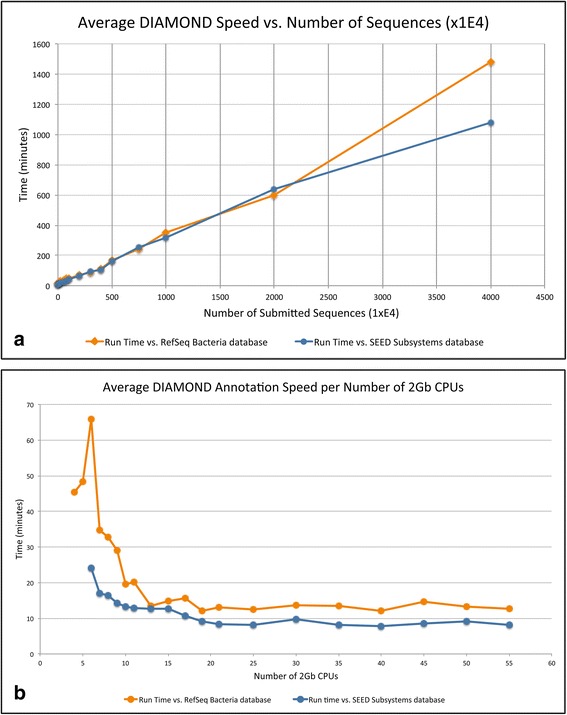
Benchmarking of SAMSA2 for resource use. **a** DIAMOND annotation time increases linearly as more input sequences are added, allowing the estimation of total annotation time. For this test, all files ran with 30 CPUs, each with 2 GB RAM. **b** Annotation speed relative to allocated memory: Higher RAM allocation allows DIAMOND to hold more of the reference databases in memory, speeding up pipeline annotation up to the point where the database is fully in memory; all files in this test contained 50,000 sequences each

Although the version 1.0 of SAMSA provided a complete metatranscriptome analysis pipeline, it depended on a public web service (MG-RAST) for the annotation step, which was a major roadblock with respect to speed due the growing popularity of this resource. In contrast, SAMSA2 is standalone in the sense that the tool dependencies are downloadable and can be run on the user’s own compute resources. SAMSA2’s annotation speed was tested with various file sizes. SAMSA2 scales relatively linearly as metatranscriptome size increases (Fig. 5a).

SAMSA2 was tested with increasing numbers of CPU and increasing RAM size. These experiments suggest that a minimum of 10 CPUs/20 GB of RAM are needed to efficiently process even small sequence files against NCBI’s non-redundant Bacteria database, approximately 6 GB (see Fig. 5b). If a larger reference database is used, more RAM will be needed.

To directly compare the runtime of SAMSA2 to SAMSA, five tiny metatranscriptomes of 50,000 sequences each were run using the two pipelines. The SAMSA2 pipeline’s average runtime was 7 h, 41 min while the SAMSA pipeline’s average runtime was 1 day, 19 h, 45 min (Table 1). Further, the SAMSA pipeline runtimes were based on a “best case scenario” in which the user has supplied metadata and granted permission to make the results available to the public immediately, resulting in the highest priority for processing. For users who wish to keep their data private, runtimes will be longer because other jobs will have higher priority. The breakdown of the pre-processing and mapping steps in SAMSA2 shows that the mapping steps are the most time consuming and the runtime depends on the database (Table 1). If a user only wants to map to the CAZy database, the total runtime with SAMSA2 is quite fast (< 30 min for 50 K sequences, Table 1). In practice with full, real metatranscriptomes on other projects (~ 50 M reads), SAMSA2 requires runtimes of several weeks whereas SAMSA1.0 runtimes are several months.

**Table 1:** Runtime of SAMSA2 components vs SAMSA (Days-Hours:Minutes:Seconds)

	SAMSA2	SAMSA2	SAMSA2	SAMSA2	SAMSA2	SAMSA 1.0
	Pre-processing	RefSeq	SEED subsystems	CAZy	Total runtime	Total runtime^a^
Average	0–0:21:10	0–5:24:18	0–0:26:16	0–0:02:49	0–6:14:21	1–19:44:00

^a^Best case scenario: Public immediately after completion (highest priority)

SAMSA2 yields consistently high accuracy scores. Using simulated metatranscriptomes made from reference reads through the use of MetaSim [17], the accuracy of SAMSA2’s matching was determined for both organism and function. Organism matching was evaluated by comparing SAMSA2’s predicted organism to the actual organism from which the starting sequence was obtained, whereas function matching was evaluated by comparing the predictive function of the entire gene sequence to the functions predicted from the smaller read sequences used as input for SAMSA2 testing. Overall, SAMSA2 had high accuracy for sequences from a selection of the most common organisms identified in gut microbiome samples, with approximately an 87.5% accurate prediction of organism at the species level. Selecting at the genus level, SAMSA2 yielded approximately 95% accuracy at the annotation step. In comparison, SAMSA genus-level accuracy on the same metatranscriptomes was 91%.

Examining annotation accuracy of functions is difficult, given the lack of standard naming conventions for functional activity of genes. To overcome this challenge, functional annotations were performed against the SEED Subsystems database, which provides a hierarchy for each function, with each specific functional annotation grouped under several more general categories. Functional annotations of reads generated by MetaSim from supplied starting sequences were annotated and then compared against the SEED Subsystems annotations of full sequences. Although there are often difficulties with predicting the exact function from partial reads, as expected (approximately 76% accuracy), the SAMSA2 pipeline overall showed excellent accuracy for predicting hierarchy at all higher levels of organization (greater than 95% accuracy). Given that other sequencing approaches generally fail to provide any functional information about the microbiome, SAMSA2’s ability to predict both organism and function for each read makes it powerful for providing a complete picture of microbial gene expression.

### Ability to customize index database

One challenge faced by nearly all bioinformatics pipelines is that some databases are not public and are restricted by institutional paywalls, whereas others may contain suspect annotations that could prove to be erroneous. Compounding this issue is the differing rate of updates to various databases; the release version of NCBI’s RefSeq database [14] used by one pipeline may be different from the version used by another similar tool, yielding differing annotation results even from the same sequencing reads as input.

Version 1 of SAMSA relied upon the databases compiled by MG-RAST. MG-RAST uses internal identifiers to combine multiple databases, including NCBI’s RefSeq, UniProt, SEED Subsystems, KEGG Orthologs, GreenGenes, SILVA SSU, and others [14, 15, 18–21]. A single internal identifier mapped to the corresponding entry for a read in each of these databases allows cross-database comparisons. Although this approach is an excellent method to compensate for a lack of a “global thesaurus” between different databases, it does not solve issues with version compatibility. MG-RAST’s version of KEGG Orthologs, for example, is the last available version before KEGG made its database private in 2011, and is thus likely out of date [22].

SAMSA2 provides a solution to the database issue by offering the ability to add or even create, custom databases that can be indexed and searched against for metatranscriptome annotation. Any database that can be downloaded in or converted to FASTA protein format, can be incorporated into SAMSA2 and used as a reference database. Downstream scripting is currently optimized to run using NCBI’s RefSeq database for organism and functional annotation, with the option to include the publicly available SEED Subsystems database [15] for hierarchical clustering of functional annotations. It is also possible to add any other available database, including more specific databases such as the CAZy database [16].

To add a custom database to the SAMSA2 pipeline, the database must be available as an unencrypted text file in FASTA format, where the header contains an internal ID, the function name, and the organism name in brackets. A single command converts this file into a DIAMOND-indexed encrypted database file, which can then be used for analysis of metatranscriptome files. Because these databases are stored on the cluster or server running SAMSA2, there is no limit to how many index databases or versions of databases can be used or consulted.

### Functional annotations and organism annotations for each input read

An important feature of metatranscriptome analysis is the ability to trace each read through the pipeline, determining exactly which sequences are assigned to a specific annotation. This tracking enables more powerful analyses, such as determining all functions performed by a specific organism or clade, determining which organisms are carrying out a specific function or category of functions, or determining the ability to evaluate potentially differing annotations from different databases.

SAMSA2 generates outfiles after each step in the pipeline, ensuring that version history and each entry read are tracked. The outfile from the annotation step contains each read’s original identifier assigned to it by the sequencing machine, as well as its organism and function annotations. Although these outfiles are later aggregated to create sorted abundance counts, additional scripting allows SAMSA2 users to alter the selection parameters for the creation of these sorted abundance counts files, specifying whether they want to include either all annotations or a subset (selecting either by organism or by functional keyword or category for sorting and selection).

By minimizing loss of information, SAMSA2 allows users to perform the longest and most computationally expensive step of annotation only once per database, after which they may return as many times as needed to the outfiles for additional analyses. There is no need to rerun the entire sample files if changes need to be made or additional reads are to be added; those can simply be aggregated on to existing results to save time and computational resources.

### Sorting of functions into hierarchical categories using SEED Subsystems

The ability to annotate mRNA sequence data from a microbial community not just to organism but also to its function is incredibly powerful. However, in such a big data context, a traditional approach, such as the results returned by a BLAST search, quickly proves overwhelming. With a single metatranscriptome likely to return anywhere from tens to hundreds of thousands of unique functions when mapped against a traditional index database such as RefSeq, determining the general activities occurring within this environment becomes next to impossible.

To overcome this issue, SAMSA2 incorporates the open-source SEED Subsystems database [15], which is uniquely organized through hierarchical classification. Each sequence in the SEED Subsystems database has a unique functional annotation but is also grouped into increasingly broad categories at four levels of hierarchy. At the highest hierarchy level, a single category may include tens of thousands of different specific functions.

Similar to other reference databases, the SEED Subsystems database may be downloaded and indexed by DIAMOND for use in annotation. Annotation against the SEED Subsystems database provides the exact functional match within the Subsystems database, but additional scripting allows the Subsystem functional hierarchy to be retrieved for each annotation. This enables analysis of the metatranscriptome at each level of hierarchy, including determination of significant differential expression and graphical representation of relative functional activity at any chosen hierarchy level (see Fig. 4). Although the change in many specific functions may be too small to be noted as significantly differentially expressed between experimental conditions, the broader category of functional activity reveals the larger scale shift in activity within the community. Full instructions are included in the SAMSA2 documentation for downloading, compiling, and using the SEED Subsystems database for examining functional hierarchies of annotations.

### Subdividing metatranscriptome data to obtain functional activity by specific organism

An important factor for microbiome analysis is the ability to perform a joint analysis of both organism and function. Without knowing which organisms are performing specific activities, any examination of microbiome activities is greatly limited. Metatranscriptomics offers the ability to determine reads matching a specific functional activity or category and which organisms produced those sequences. SAMSA2 provides this information by offering optional scripts to segment the data by organism or by function or functional category. Results from an entire microbiome may be subdivided to examine only the activities of reads annotated to a single genus or species, and functional annotations in a specific group may be examined to determine which organisms produce those transcripts. By matching the read IDs assigned to transcripts against the reference databases, which contain both organism and functional annotations, it is possible to obtain both the organism and functional results for each specific read in the entire metatranscriptome.

## Discussion

The study of complex microbial environments, which may contain many different interacting organisms, requires large amounts of data to fully understand. Current approaches, such as 16S rRNA sequencing, can provide a broad overview of which groups of microbes are present in an environment, but they fail to offer enough resolution to differentiate between closely related genera or species, and they provide no information about potential activities being performed by members of the microbiome. Although metatranscriptomics generally requires higher initial sample quality and has higher costs in sequencing and processing, it offers detailed insights into both the organisms present and their current transcriptional activity.

Version 1.0 of SAMSA was intended for use by life science researchers who had limited command line and coding experience, providing straightforward, completely open source tools for the analysis of metatranscriptomic datasets. Many researchers don’t have access to a local computing cluster or on-demand compute resources; SAMSA 1.0 allows these researchers to analyze the large metatranscriptome data files by outsourcing the heavy lifting of annotation to the external MG-RAST server. Downstream SAMSA 1.0 programs condense and reduce this huge amount of information and can run on a personal computer.

We created the updated SAMSA2 pipeline to be faster and more customizable than SAMSA 1.0 through use of on-demand compute resources; the pipeline still uses the same general format for analyzing metatranscriptomes. By switching to locally installed, well-established and trusted dependencies that take advantage of the increased power of a supercomputing instance, SAMSA2 offers faster analysis and provides more options for in-depth examination. Additionally, the ability to add custom databases that may be indexed and used as references allows SAMSA2 to be upgraded when newer database versions become available without having to reinstall the entire pipeline. Version control ensures that previous analyses may be repeated without concern over reference database version. The advantages of SAMSA2 are summarized in Table 2.

**Table 2:** Summary of differences between SAMSA versions

Advantage	SAMSA2	SAMSA 1.0
Runtime^a^	Weeks	Months
Accuracy	95% (genus level)	91% (genus level)
Custom database options	Yes	No
Version control for reproducibility	Yes	No
Data slicing	Organism, group, function, taxonomy level, functional category	Organism, function
Figures and graphs	PCA, pie, barplot, diversity	barplot

^a^Runtime dependent on number of reads. SAMSA 1.0 runtimes are longer if permission is not given to MG-RAST to make the data public immediately

An alternate method of metatranscriptomics analysis is to first assemble reads into longer continguous sequences, or “contigs,” before mapping to sequence databases [23–26]. This method is more difficult, more likely to result in chimeric contigs, and more likely to miss low-expressing genes, although it may be more appropriate for applications in which strain-level taxonomy information is needed. The method employed by SAMSA and other tools is to directly map the reads to protein databases. The SAMSA2 pipeline is appropriate for biologists or bioinformaticians who want to quickly assess genus-level gene expression and functional annotations of genus-level taxa or whole communities. SAMSA2 aims to be one potential pipeline option with the flexibility to adapt to the needs and resources of a wide range of biological investigations.

## Conclusion

We have developed SAMSA2, a complete metatranscriptome analysis pipeline that enables rapid, customizable annotation and analysis of metatranscriptome data. SAMSA2 can be installed on a local or cloud server and supports incorporation of multiple reference databases, including NCBI’s RefSeq for organism and specific function identification, and SEED Subsystems for hierarchical ontologies of functional activity groups. SAMSA2 is open source (https://github.com/transcript/samsa2).

## Availability and requirements

### Project name: SAMSA2

**Table:** 

Project home page: https://transcript.github.io/samsa2/	
The repository provides the necessary pipeline scripts for SAMSA2, example data files, and example workflows to demonstrate their use. Links are also provided for installation of underlying programs and requirements as listed below.	
Operating system(s): any supporting Python 2.7 (tested on Linux)	
Programming language(s): Python 2.7, R 3.0	
Programs: DIAMOND=0.8.38, Trimmomatic=0.36, PEAR=0.9.8, SortMeRNA=2.1	
R packages: DESeq2 [1.12.3], pheatmap [1.0.8], ggplot2 [2.1.0], RColorBrewer [1.1.2], reshape2 [1.4.1], data.table [1.9.6], knitr [1.13], vegan [2.4.0]	
License: The GNU General Public License, version 3 (https://www.gnu.org/licenses/gpl-3.0.en.html)	
The datasets analyzed during the current study are available in the SAMSA2 repository, available on Github and provided in the repository.	

## Abbreviations

DIAMOND: a BLAST-like sequence aligner that uses translated sequence queries to greatly increase annotation speed over traditional BLAST
MG-RAST: the MetaGenomics Rapid Annotation using Subsystems Technology server, a public analysis pipeline for handling metagenome and metatranscriptome datasets
mRNA: messenger RNA, single-stranded genetic material that codes for the creation of proteins
PCA: principal component analysis
SAMSA: Simple Analysis of Metatranscriptomes through Sequence Annotation the documented pipeline
SEED: a protein database that seeks to group sequences into hierarchical categories, created by the Fellowship for Interpretation of Genomes (FIG group)
